# Current Advancements in Sactipeptide Natural Products

**DOI:** 10.3389/fchem.2021.595991

**Published:** 2021-05-20

**Authors:** Yunliang Chen, Jinxiu Wang, Guoquan Li, Yunpeng Yang, Wei Ding

**Affiliations:** ^1^State Key Laboratory of Microbial Metabolism, Joint International Research Laboratory of Metabolic and Developmental Sciences, School of Life Sciences and Biotechnology, Shanghai Jiao Tong University, Shanghai, China; ^2^School of Agricultural Equipment Engineering, Jiangsu University, Zhenjiang, China; ^3^Northwest Institute of Eco-Environmental and Resources, Chinese Academy of Sciences, Lanzhou, China; ^4^Chinese Academy of Sciences Center for Excellence in Molecular Plant Sciences, Shanghai Institute of Plant Physiology and Ecology, Chinese Academy of Sciences, Shanghai, China

**Keywords:** sactipeptide, antibaceterial, thioether, RiPPs: ribosomally produced and post-translationally modified peptides, SAM radical enzyme

## Abstract

Ribosomally synthesized and post-translationally modified peptides (RiPPs) are a growing class of natural products that benefited from genome sequencing technology in the past two decades. RiPPs are widely distributed in nature and show diverse chemical structures and rich biological activities. Despite the various structural characteristic of RiPPs, they follow a common biosynthetic logic: a precursor peptide containing an N-terminal leader peptide and a C-terminal core peptide; in some cases,a follower peptide is after the core peptide. The precursor peptide undergoes a series of modification, transport, and cleavage steps to form a mature natural product with specific activities. Sactipeptides (Sulfur-to-alpha carbon thioether cross-linked peptides) belong to RiPPs that show various biological activities such as antibacterial, spermicidal and hemolytic properties. Their common hallmark is an intramolecular thioether bond that crosslinks the sulfur atom of a cysteine residue to the α-carbon of an acceptor amino acid, which is catalyzed by a rSAM enzyme. This review summarizes recent achievements concerning the discovery, distribution, structural elucidation, biosynthesis and application prospects of sactipeptides.

## Introduction of Typical Sactipeptides

**Figure 1:**
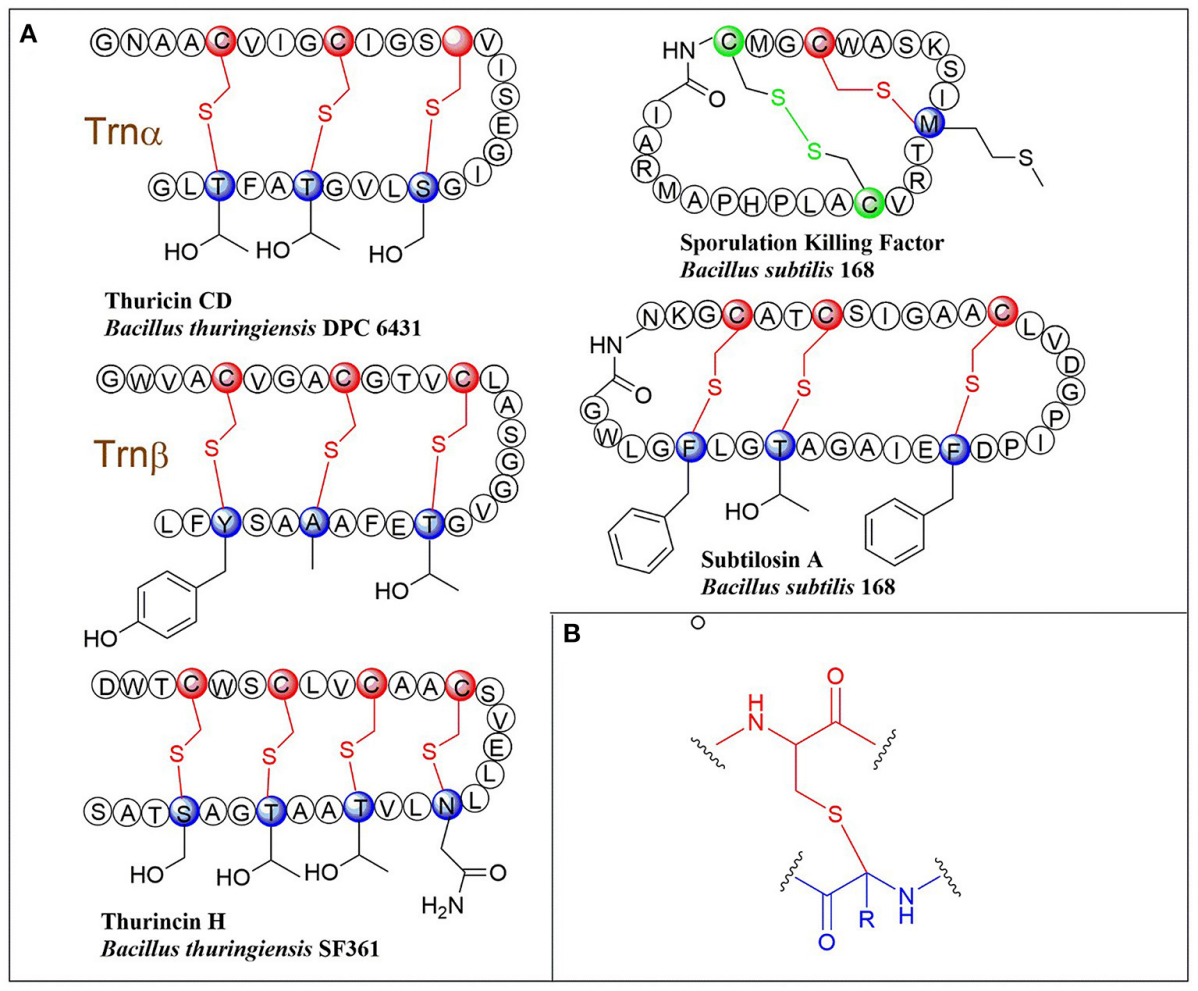
**(A)** Schematic representation of early-discovered sactipeptides from *Bacillus*. The donor and acceptor residues for thioether linkage marked with red and blue respectively, the thioether linkage marked with red, the residues and disulfide bond marked with green. **(B)** The detailed structure of thioether linkage.

**Figure 2:**
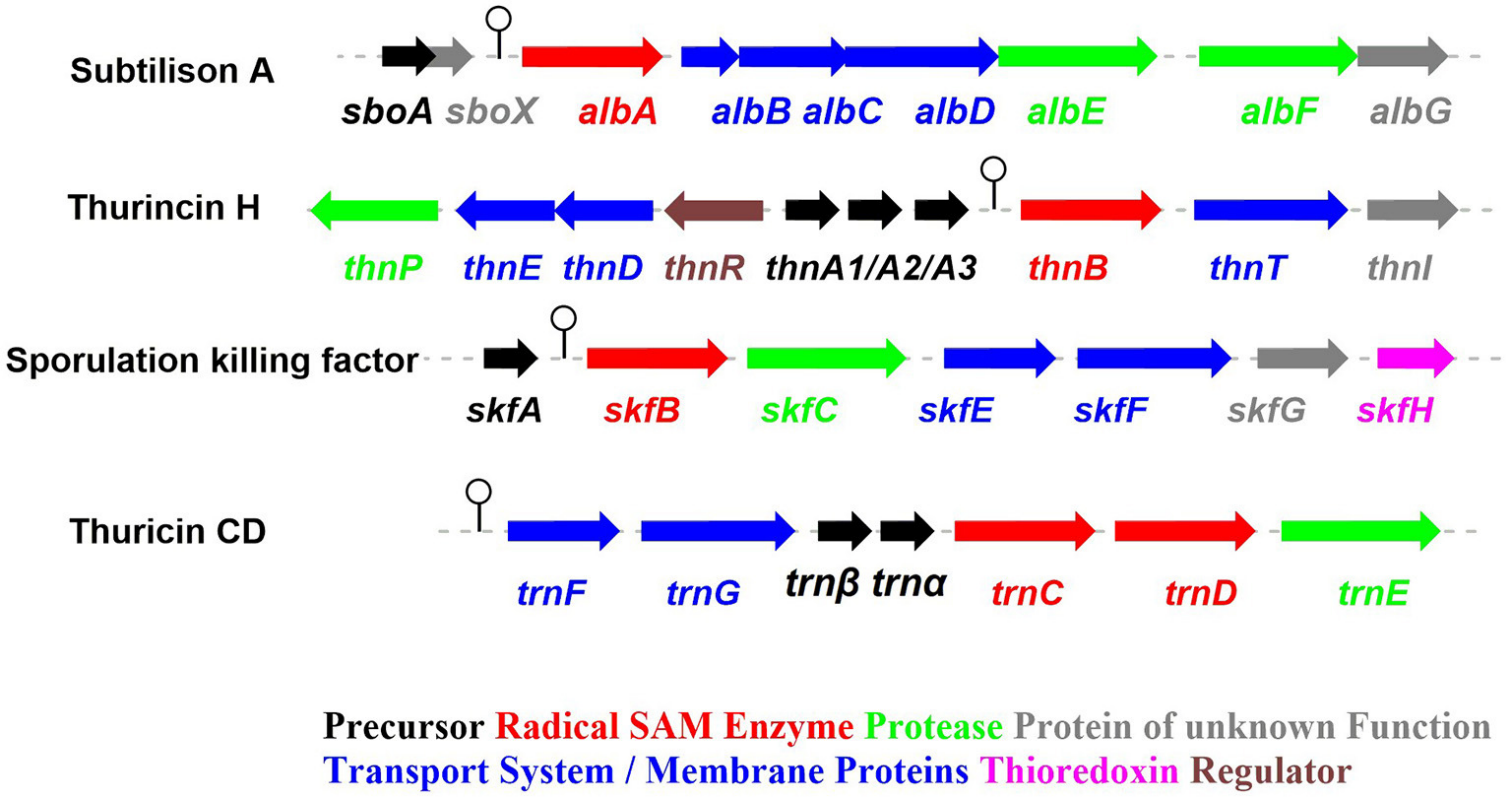
The biosynthetic gene clusters of the early-discovered sactipeptides. Subtiliosn A, Thurincin H, Sporulation killing factor (SKF), Thuricin CD (Fluhe and Marahiel, 2013).

Ribosomally synthesized and post-translationally modified peptides (RiPPs) are a major class of natural products found in all three domains of life. They possess vast structural and biological diversity, representing a promising source for new antibiotics and other drugs. A small but growing family of RiPPs is sactipeptides, which contain one or more sactionine residues characterized by intramolecular thioether linkage between a Cys sulfur and the a-carbon of another amino acid. Seven sactipeptides have been characterized to date. According to chronological order of discovery, they are subtilosin A from *Bacillus subtilis* 168 (Kawulka et al., 2003, 2004), thurincin H from *Bacillus thuringiensis* SF361 (Lee et al., 2009), sporulation killing factor (SKF) from various *Bacillus subtilis* (Engelberg-Kulka and Hazan, 2003), the two-component thuricin CD (Trn-α and Trn-β) from *Bacillus thuringiensis* DPC6431 (Rea et al., 2010), thuricinZ/Huazacin from *Bacillus thuringiensis* serovar *huazhongensis* (Hudson et al., 2019; Mo et al., 2019), ruminococcin C1 from *Ruminococcus gnavus* E1 (Balty et al., 2019) and newly identified streptosactin from *Streptococcus* spp. The early six members (Figures 1, 2) were all from *Bacillus*. Subtilosin A is the first discovered sactipeptide in 1985 (Babasaki et al., 1985). Until 2003, its unusual structure was solved and comprised three thioether bridges by NMR spectroscopy (Kawulka et al., 2003). In 2004, further studies confirmed the three amino acids' configurations involved in thioether bond formation as L-Phe22, D-Thr28, and D-Phe31, while thought all as L-type initially (Kawulka et al., 2004). Thurincin CD, a two-component antimicrobial peptide, is the second structural elucidated sactipeptide. The two-component of Thurincin CD Trn-α and Trn-β both have three thioether linkages. Their six cycles locate between amino acid residue Cys5 to Thr25, Cys9 toThr25, Cys13 to Ser21 in Trn-α or Cys5 to Tyr28, Cys9 to Ala25, Cys13 to Thr21 in Trn-β (Rea et al., 2010). One year later, the NMR data and structure calculations elucidated that Trn-α has L-stereochemistry at Ser21 (α-R) and Thr25 (α-R) and D-stereochemistry at Thr28 (α-S), they form an LLD isomer. The Thr21 (α-R) and Ala25 (α-R) of Trn-β are L-configuration, but Tyr28 (α-R) is D-configuration; they also form an LLD isomer in Trn-β (Sit et al., 2011a). The sporulation killing factor (SKF) is 26 amnio acids cyclic peptide with two intramolecular crosslinks using isotope labeling and high-resolution tandem mass spectrometry. One is a thioether bond between a cysteine and the α-carbon of a methionine, and the second is a disulfide bond. The NMR elucidated that Cys4 is linked to the α-carbon of Met12, which is the characteristic of sactipeptide (Liu et al., 2010). Similarly, the four intramolecular thioether linkages of thurincin H between Cys4 to Ser28, Cys7 to Thr25, Cys10 to Thr22, Cys13 to Asn19 are all determined to be D-configurations (Sit et al., 2011b). Qi et al., utilized deuterated solvent and NiCl2/NaBH4 reduction reaction that products were subjected to HRMS/MS analysis (Mo et al., 2019). Comparing characteristic fragment ions indicated deuterium incorporation into Asp26, Thr30, Tyr34, and His38 which are the receptor of the four sactionine rings. The study also confirmed that the radical-SAM-dependent thioether crosslinks in thuricin Z biosynthesis are at Cα atoms, not at Cβ atoms (Duarte et al., 2018; Mo et al., 2019). Durate and coworkers have shown that ruminococcin C1 contains four sulfur to α-carbon thioether cross-links between Cys3 and Asn16, Cys5 and Ala12, Cys22 and Lys42, and Cys26 and Arg34. Calculations with combined assignment and dynamics algorithm for NMR applications (CYANA) software identified the four α-carbon atoms of thioether bridge residues are all D-stereochemistry (DDDD stereoisomer). Therefore, NMR and HRMS/MS integrated with chemical derivatization usually can give the primary structure and stereochemistry of sactipeptides; the further NMR datas also uncover the dimensional structures α-carbon atoms of thioether bridge such as LDD in subtilosinA, LLD in thuricin CD and DDDD in thurincinH respectively (Chiumento et al., 2019; Roblin et al., 2020). The latest discovered streptosactin is the first sactipeptide from *streptococcus*. The mature natural streptosactin is prohibitively low yield for NMR confirmation. Bushin and coworkers produced putative mature product with heterologous co-expression of the precursor peptide and modification enzyme genes in *Escherichia coli* and compared its chromatographic and HRMS/MS properties with the natural streptosactin isolated from the fermentation broth. Comparing with authentic standard, it indicated that streptosactin is a 14mer peptide with two S-Cα bonds between Cys15 and Ser28, Cys31 and Gly34 (Bushin et al., 2020). Despite recent structural studies of sactipeptides, the exact mechanism of thioether cross-links (sactionine) formation remains to be uncovered.

## Biosynthesis of Sactipeptides

**Figure 3:**
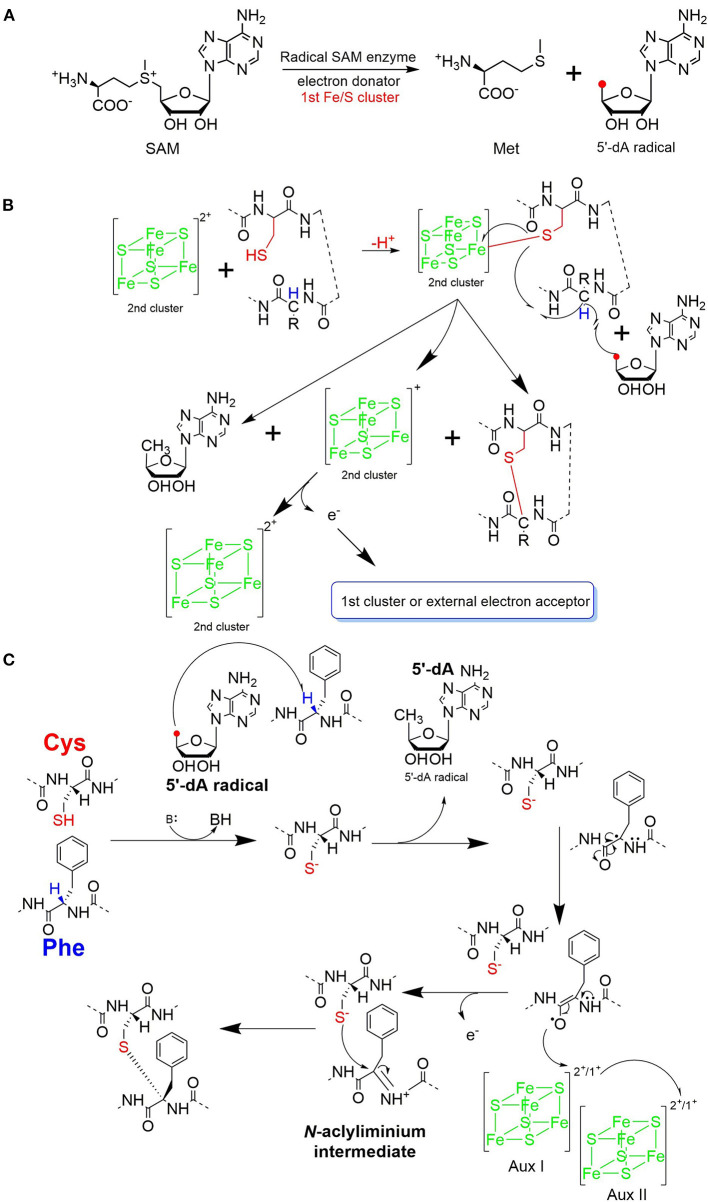
Proposed mechanisms for thioether bond formation in sactipeptide biosynthesis (Mahanta et al., 2017). **(A)** Reductive SAM cleavage reaction results in methionine and the 5′-deoxyadenosyl radical. **(B)** A proposed mechanism for thioether bond formation. **(C)** Another proposed mechanism for thioether bond formation. After H-atom abstraction from the Cα-atom, the carbon-centered radical is oxidized to produce an N-acyliminium intermediate. This intermediate then reacts with the thiol group of a cysteine residue, resulting in a Cα-thioether bond. The auxiliary clusters I and II are proposed to serve as an electron conduit.

The process of sactipeptide biosynthesis is similar to the general biosynthetic logic of RiPPs. Firstly, the sactionine synthase generally recognizes the N-terminal leader peptide sequence to bind the corresponding precursor peptide. The C-terminal of the precursor peptide (core peptide) is post-translationally modified by sactionine synthase to form S-Cα thioether bonds. After that, the leader peptide is removed to form the final mature sactipeptides (Mcintosh et al., 2009; Arnison et al., 2013; Cotter et al., 2013). In addition to the precursor peptide and modification enzyme genes, the sactipeptide biosynthetic gene cluster generally includes genes such as proteases, membrane proteins/transport systems, regulatory factors, and thioredoxin (Bruender et al., 2016). Based on the fact that at least one radical SAM (rSAM) enzyme is encoded in each of the sactipeptide biosynthetic gene clusters, such enzymes may play a crucial role in catalyzing the formation of thioether bonds (Fluhe and Marahiel, 2013). rSAM enzyme usually contains a characteristic CX_3_CX_2_C motif ligating a [4Fe-4S] cluster (Atta et al., 2012; Fluhe et al., 2013). The sulfydryls of three cysteines bind three irons of the [4Fe-4S] cluster. The fourth iron is bidentate with a SAM, which is then reductively cleaved by the radical enzyme into a 5′-deoxyadenosyl (5′-dAdo) radical and methionine (Figure 3A) (Sofia et al., 2001; Frey et al., 2008; Roach, 2011). The generated 5′-dAdo radical specie can trigger various chemical reactions (Pujol et al., 2011; Fluhe et al., 2013; Wieckowski et al., 2015; Ding et al., 2016; Mozolewska et al., 2017) and form various natural compounds. Besides the conservative [4Fe-4S] cluster, sactionine synthases usually harbor auxiliary one or two iron-sulfur clusters. These enzymes belong to a growing subclass of radical SAM enzymes with SPASM domain (Aux I and AuxII) or twitch domain (Aux I) (Lanz and Booker, 2012, 2015; Grell et al., 2015b). The first member of this subset is the anaerobic sulfatase-maturating enzyme (anSME) which is responsible for the post-translational modification of sulfatase with catalyzing cysteine or serine residue to a Cα-formylglycine (Benjdia et al., 2007). Crystal structure investigations of anSME indicated that it coordinates two additional [4Fe-4S] clusters in its SPASM domain (Benjdia et al., 2009, 2011; Goldman et al., 2013). The first structure elucidation of sactisynthase was CteB. It has the (β/α)6-TIM barrel fold which is characteristic of radical SAM enzymes, as well as a C-terminal SPASM domain containing two auxiliary [4Fe-4S] clusters. One [4Fe-4S] cluster in the SPASM domain of CteB exhibits an open coordination site in the absence of peptide substrate, which is coordinated by a peptidyl-cysteine residue in the bound state. The crystal structure of CteB also shows a RiPP precursor peptide recognition element (RRE) in the N-terminal domain, which has high structural similarity to the recently discovered motif present in several RiPP modification enzymes. This first sactisynthase structure sheds light on structures and mechanisms of other members of this class such as AlbA or ThnB (Grove et al., 2017). Although all sactionine synthases have auxiliary [4Fe-4S] clusters similar with anSME, the exactly catalytic mechanism and the functions of additional iron-sulfur clusters are still less understood.

### AlbA in Subtilosin Biosynthesis

Subtilosin A is macrocyclic peptide with 35 residues, and its biosynthetic enzymes are encoded by the *sbo-alb* locus of *Bacillus subtilis* 168 (Figure 1) (Kawulka et al., 2004). *In vitro* studies, AlbA is a rSAM enzyme that contains three [4Fe-4S] clusters. It catalyzes sactionine formation on the linear precursor peptide SboA encoded by gene *sboA*. This modification is determined by the sequence of leader peptide and the RRE domain (RiPP precursor peptide *R*ecognition *E*lement) in AlbA (Fluhe et al., 2012). The Cys residues (Cys129, Cys133, and Cys136) mutated to Ala residue in the first [4Fe-4S] cluster of AlbA would ultimately lose the function of cleaving the SAM into methionine and a 5'-dAdo. It was speculated that AlbA harbors two auxiliary [4Fe-4S] clusters in its C- terminal, the same with familiar rSAM enzymes PqqE, anSMEs, and MftC, which are involved in the biosynthesis of pyrroloquinoline quinone, anaerobic sulfatase, and Mycofactocin maturation, respectively. This extension region in rSAM enzymes for binding iron-sulfur clusters named as “SPASM” domain (Grell et al., 2015a). When the Cys residues binding the auxiliary iron-sulfur cluster were mutated, the mutant enzymes were still able to cleave SAM, but lose capacity of thioether bond formation. Ten AlbA mutants were made to investigate its tolerance and region preference in construction of the thioether bond, that is three donor cysteine residues (C4, C7, C13) singly mutated into alanine and serine respectively, the four acceptor residues point mutated to alanine or phenylalanine respectively (F22A, T28A, F31A, F31Y). *In vivo* studies showed that neither SboA's mutants carrying an alanine nor a serine as donor amino acid could not be a substrate for AlbA. Furthermore, AlbA cannot catalyze the thioether bond formation and subtilosin A variant production when acceptor amino acids mutated to alanine (F22A, T28A, and F31A) (Fluhe et al., 2012). A SboA mutant carrying an acceptor amino acid Tyr to Phe can be catalyzed by AlbA, indicating AlbA has a limited tolerance of structural similar amino acid at the thioether acceptor site. On the other hand, a mechanism of AlbA catalyzed thioether bond formation has been investigated (Figures 3A,B). AlbA directly catalyzes the hydrogen atoms abstraction on the α-carbon atom of the acceptor amino acid to form a ketoimine intermediate; then the thiol of the donor amino acid nucleophilic attacks α-carbon atom of ketoimine intermediate to create the thioether bridge (Figure 3C) (Benjdia et al., 2016, 2017; Mahanta et al., 2017). AlbA is very similar to anSME in biochemical function and structure. Based on the biochemical and structural studies, anSME was shown to catalyze the H-atom abstraction of β-carbon cysteine, leading to a C-S unsaturated bond formation between the Cβ-atom and the deprotonated thiol group of its cysteine substrate. It speculated that the SPASM [4Fe−4S] centers of anSME play a critical redox function (Goldman et al., 2013). Even more, the successful solid-phase synthesis of a bicyclic dipeptide containing thioether bonds through a N-acyliminium intermediate indicated the feasibility of the nucleophilic addition mechanism as the case of AlbA (Nielsen et al., 2005; Benjdia et al., 2016). Moreover, the auxiliary [4Fe−4S] of AlbA was estimated to participate in the radical intermediate oxidation as proposed for anSME, which remains to be fully elucidated in the future.

### SkfB in Sporulation Killing Factor Biosynthesis

The sporulation killing factor short as SKF is a 26 amino acids sactipeptide (Figure 1) that plays a key role during the strain's sporulation (Gonzalez-Pastor et al., 2003; Velho et al., 2013). SKF contains a thioether bond cross-link between Cys4 and Met12, a disulfide bond cross-link between Cys1 and Cys16, and a head-to-tail cyclic architecture by an amide bond. The *skf* gene cluster is encoding the enzymes for SKF biosynthesis. It comprised of a precursor gene *skfA*, post-modification genes *skfB* (rSAM enzyme), *skfC* (putative protease), *skfH* (putative thioredoxin), *skfE/skfF* for export/resistance, and *skfG* for unknown function (Figure 2) (Velho et al., 2013). Like AlbA, SkfB is the functional subclass rSAM enzyme responsible for sactionine formation (Fluhe et al., 2013). Through mutabiosynthesis studies, it indicated that SkfB has an individual tolerance on the donor and acceptor amino acid residues of SkfA. Furthermore, although SkfB strictly recognizes the donor residue Cys4 of SkfA, for the acceptor amino acid in SkfA, SkfB has moderate tolerance for the mutants carrying hydrophilic amino acids instead of Met12, but a better tolerance for substitutions with hydrophobic amino acids, but most tolerance for substitutions with hydrophobic amino acids. Therefore, SkfB is also leader peptide dependent rSAM enzyme. Like all radical SAM enzymes, all of the conservative cysteine coordinated to radical SAM [4Fe-4S] cluster of SkfB were essential for cleaving SAM into 5'-dAdo radical and thioether cross-link formation, the absence of auxiliary iron-sulfur cluster do not affect 5'-dAdo radical abstracting H-atom of Cα but thioether bond formation (Bruender and Bandarian, 2016; Kincannon et al., 2018). Futhermore, the first crystal structure elucidation of enzymes containing auxiliary iron-sulfur is anSME; for sactisynthase, CteB is the first and SkfB the second (Grove et al., 2017; Grell et al., 2018). Structural data shows that SkfB has an N-terminal RiPP recognition element (RRE) domain for peptide-binding. Subsequent is a classic partial (β/α)_6_ barrel structure bounding the [4Fe-4S] cluster, and the C-terminal is a twitch domain harboring a [2Fe-2S] cluster, which is the same with the pyrroloquinoline quinone biosynthesis enzyme PqqE (Grell et al., 2018; Zhu et al., 2020). Unambiguously, SkfB catalyzes hydrogen abstraction from the α-carbon of Met12 of precursor peptide SkfA (Bruender and Bandarian, 2016). Hydrogen's abstraction from α-carbon of donor amino acid has been verified uniform in the initiating catalysis by sactisynthase, but the mechanism for thioether linkage formation is ambiguous. Therefore, the role of the auxiliary [2Fe-2S] cluster in SkfB needs to further investigate.

### ThnB in Thurincin H Biosynthesis and TrnC/D in Thuricin CD Biosynthesis

Thurincin H (Figure 1) is a 31-residue sactipeptide from *B. thuringiensis* SF361 (Sit et al., 2011b). Its biosynthetic gene cluster *thn* comprises three identical copies gene *thnA1-A3* encoding three of the same 40 amino acids precursor peptide, an rSAM enzyme gene *thnB*, a protease *thnP*, three-component ABC transporter gene *thnD/E/T*, transcriptional regulator gene *thnR*, and an unknown function gene *thnI* (Figure 2). Biochemical studies on ThnB indicated that it was the functional enzyme for sactionine bond formation (Wieckowski et al., 2015). Like SkfB, ThnB coordinate a characteristic radical SAM [4Fe-4S] and an auxiliary [4Fe-4S] cluster (twitch domain). Amino acid substitution studies indicated that the first rSAM [4Fe-4S] cluster is coordinated to Cys165, Cys169 and Cys172 of ThnB and is essential for SAM cleavage and 5'-dAdo radical formation, while Cys449, Cys455, and Cys458 putatively coordinate a second [4Fe-4S]. Although the variant ThnB mutants that lacked the N-terminal RRE domain was incapable of recognizing the precursor peptide ThnA1-A3 and forming the thioether bond, they still keep the activity for cleaving SAM to 5′- dAdo radical. This result suggested that the RRE domain in ThnB participates in the interaction with ThnA (Burkhart et al., 2015; Wieckowski et al., 2015). Considering that ThnB is one member of the SPASM/Twitch rSAM enzyme subclass, it was hypothesized that its catalytic mechanism is similar to other members of the SPASM subclass sactisynthase, such as AlbA and SkfB (Figure 3). Thuricin CD (Figure 1) is a two-component antimicrobial sactipeptides with 30 residues produced by *Bacillus thuringiensis* DPC 6431 (Rea et al., 2010); they have distinct structures and named as Trnα and Trnβ. This pair sactipeptides showed good activity against *Clostridium difficile* when there were incubated together in the assay. In addition to the precursor peptides, the thuricin CD biosynthetic gene cluster encodes five proteins: rSAM enzyme TrnC and TrnD which have the same function but with very low homology, unknown function enzyme TrnE, ABC transporter TrnF and TrnG (Figure 2) (Abicht et al., 2012; Mathur et al., 2017). Nevertheless, further studies are needed to identify the indeed mechanism of TrnC and TrnD involved in thuricin CD biosynthesis.

### RumMC in Ruminococcin C (RumC) Biosynthesis

**Figure 4:**
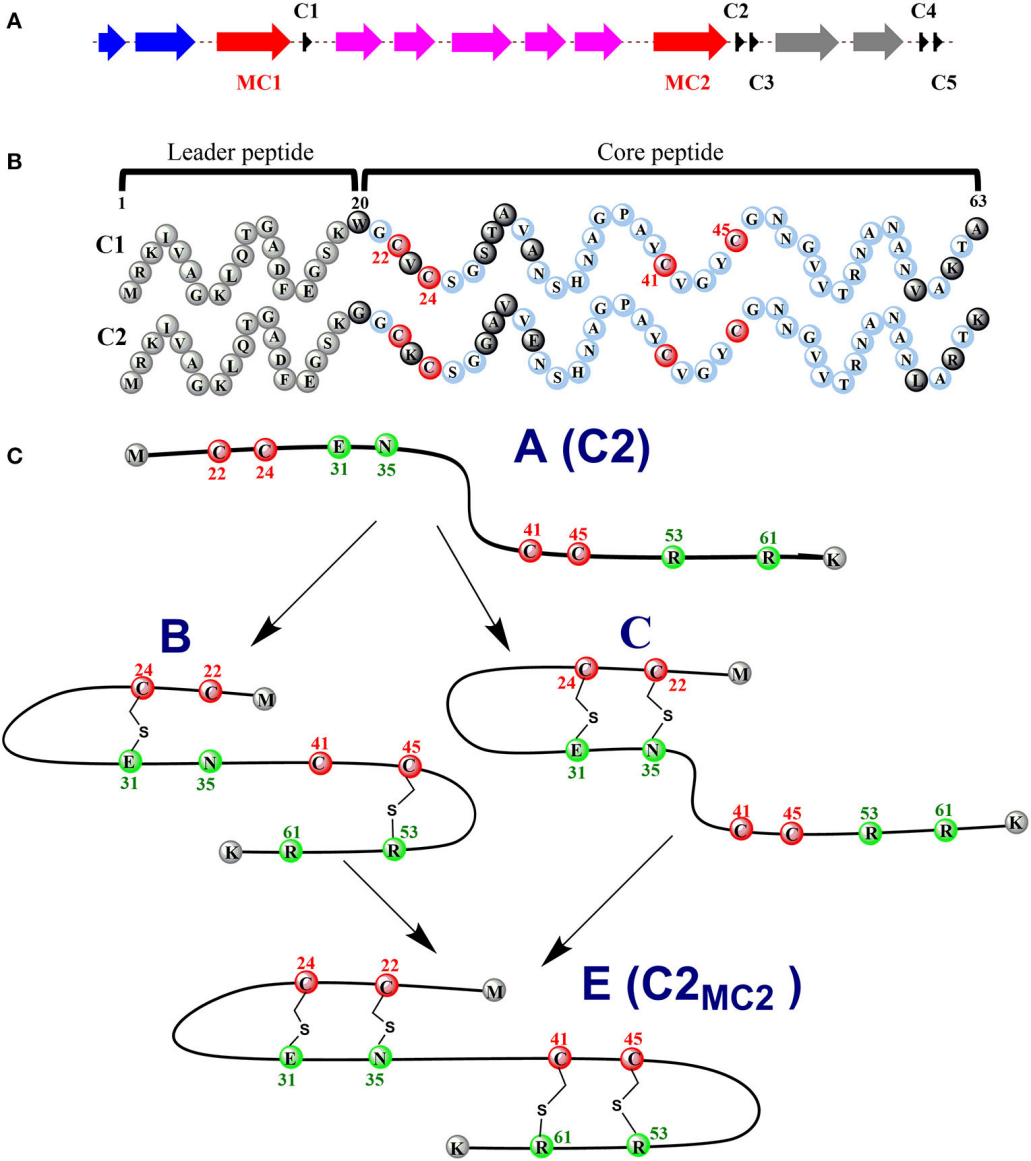
Expression of the C1/C2 peptide alone or with the radical SAM enzyme MC1/MC2 (C1MC1/ C2MC2) in *E. coli*. **(A)** the gene cluster involved in RumC biosynthesis. *Black, c1* to *c5*, genes predicted to encode RumC precursor peptides; *red, mc1* and *mc2*, genes predicted to encode tailoring radical SAM enzymes; *purple*, genes predicted to be involved in immunity; *blue* and *gray*, putative exporters. **(B)** sequences of the C1 and C2 peptides. Amino acid residues from the predicted leader sequence are in *gray*. Conserved amino acid residues from the core sequence are in light *blue*, and non-conserved amino acid residues are in *black*. The four conserved cysteine residues are in *red*. *Numbers* indicate the relative position to the sequence. **(C)** proposed sequential order for the formation of thioether bridges in RumC2. Starting from precursor A, the first thioether bridges (i.e., the Cys24-Glu31 and Cys45-Arg53 bridges) are installed in each hairpin domain (intermediate B). Then the second thioether bridges, Cys22-Asn35 (intermediate C) and Cys41-Arg61, are formed, leading to the production of the mature peptide C2MC2 (species E) with two hairpin domains (Balty et al., 2019, 2020).

Ruminococcin C (RumC) is a new type of sactipeptide discovered in the human microbiota (Crost et al., 2011). The RumC biosynthetic gene cluster contains a complicated set of gene duplications and rearrangements (Pujol et al., 2011) (Figure 4A). It is worth noting that the gene cluster includes five precursor peptide genes *c1* to *c5*, which are 63 amino acids in length and similarity between 70 and 87% each other. Four strictly conserved Cys residues and a highly conserved C-terminal region are important for these polypeptides (Figure 4B). RumMC1 and RumMC2 are two modification enzymes in the gene cluster. These two enzymes show a fantastic sequence identity (>95%) and each possess two conserved cysteine motifs: CX3CX2C and CX13GX4CX36CX2CX5CX2CX18C. The first cysteine motif is the classic [4Fe-4S] bonding domain in rSAM enzymes, and the second is the characteristic of a large subclass rSAM enzymes with SPASM domain. Co-expressions of rSAM enzymes RumMC1 and RumMC2 in *Escherichia coli* were carried to study tailoring reactions for thioether bond formation toward precursor peptide C1 and C2. Co-expressing *c1* and *mc1, c2* and *mc2* obtained two new peptides with high purity, named C1MC1 and C2MC2, respectively. Mass spectrometry revealed that the molecular weight of C1MC1 and C2MC2 were 8-dalton less than the corresponding linear peptides. It suggested that four thioether bonds formation in the molecule. Subsequently, the researchers further analyzed C1MC1 and C2MC2 by tandem mass spectrometry and identified C1MC1 modification sites as Ala31, Asn35, Arg53, and Lys61; The C2MC2 modification sites are Glu31, Asn35, Arg53, and Arg61. Considering the high sequence similarity between the two precursor peptides and the two modification enzymes, the researchers performed cross-co-expression experiments on the two groups of precursor peptides and modification enzymes. *In vivo* experiments show that MC1 cannot modify C2, but MC2 can effectively modify C1. Interestingly, C1MC2 and C1MC1 have the same spectroscopic characteristics, suggesting that the two peptides contain the same post-translational modification. Subsequently, the researchers took three mutants of C2 (C2_A22A24_, C2_A41A45_, and C2_A24_) as the substrates and performed *in vitro* catalytic experiments with rSAM enzyme MC2. A series of mass spectrometry detections determined that the thioether bonding formation position of C2MC2 was Cys22-Asn35, Cys24-Glu31, Cys41-Arg61, and Cys45-Arg53, respectively. Unlike the previously identified sactipeptides, RumC2 contains two hairpin structures, Cys22 and Cys24 are connected to Asn35 and Glu31 to form the first hairpin structure, Cys41, Cys45 are connected to Arg53 and Arg51 to form the second hairpin structure (Figure 4C). The catalytic assay of the N-terminal truncation of C2 (C2_28−63_) and its mutants (C2_28−63_A41, C2_28−63_A45) as substrates showed that the sequential order of thioether bond formation in the two hairpin structure; The inner Cys45-Arg53 linkage is earlier formed than the outer Cys41-Arg61 linkage. Further enzymatic kinetics showed that the formation of thioether bridges in the two terminal regions of the precursor peptide was relatively independent. The formation of thioether bridges proceeded from the N-terminal direction to the -C-terminal direction. The isotope-labeled hydrochloric acid (DCl) hydrolysis experiments confirmed that the four acceptor amino acid residues for the formation of thioether bonds are all L-configuration (Balty et al., 2019). Sactisynthases usually have substrate tolerance for the acceptor amino acid. RumMC2 catalyzed C2_28−63_-G61 peptide to form two doubly bridged products, except the crosslink in the natural sit. Interestingly, it can catalyze the thioether formation in an unnatural position, which is the first case in sacisythnase; only the rSAM epimerase PoyD can epimerize the amino acid in unnatural positions (Parent et al., 2018). This data suggests that the side chain of the acceptor amino acid residue and other unknown factors likely give the correct installation of sactionine in sactipeptides. Investigation of the mechanism of sactisynthase in ruminococcin C showed that RumMC2 is a typical SPASM domain rSAM enzyme that harbors three [4Fe-4S] clusters depending on UV-visible spectra and EPR data. Besides the radical SAM [4Fe-4S] cluster, three cysteines in RumMC2 SPASM domain coordinate the portion of AuxI [4Fe-4S] cluster, and another four cysteine residues wholly coordinate the AuxII [4Fe-4S] cluster. The fourth ligand of AuxI [4Fe-4S] cluster is unclear. Notably, H-atom abstraction to form a carbon radical is a route chemistry for thioether bridge formation. MS results of the intermediacy species, supporting the mechanism that substrate radical could undergo rearrangement into a ketoimine intermediate, which is then nucleophilic addition by a sulphydryl of the cystein to form a thioether bridge. This pattern is the same as the AlbA in subtilosin A biosynthesis. During the same year, Duarte et al. also reported their similar research results on RumC1. They produced large-scale 13C- and 15N-labeled mature RumC1 by heterologous expression and used NMR to analyze the structure(Chiumento et al., 2019). These data indicated that RumC1 has four S-Cα thioether bonds, folding itself into a double hairpin-like motif. This characteristic is unique within the currently reported sactipeptide family; The acceptor amino acids Ala12, Asn16, Arg34, and Lys42 of the sactionines in RumC1 are D-configurations which are opposite to a recent report by Berteau and coworkers. The compact double hairpin-like structure gives RumC1 a high tolerance with pH from 2 to 11 and even temperature from 70 to 100°C; These advantages are facilitated with the pharmaceutical industry (Balty et al., 2020; Roblin et al., 2020).

### *thzC/D* in Thuricin Z/Huazacin Biosynthesis

**Figure 5:**
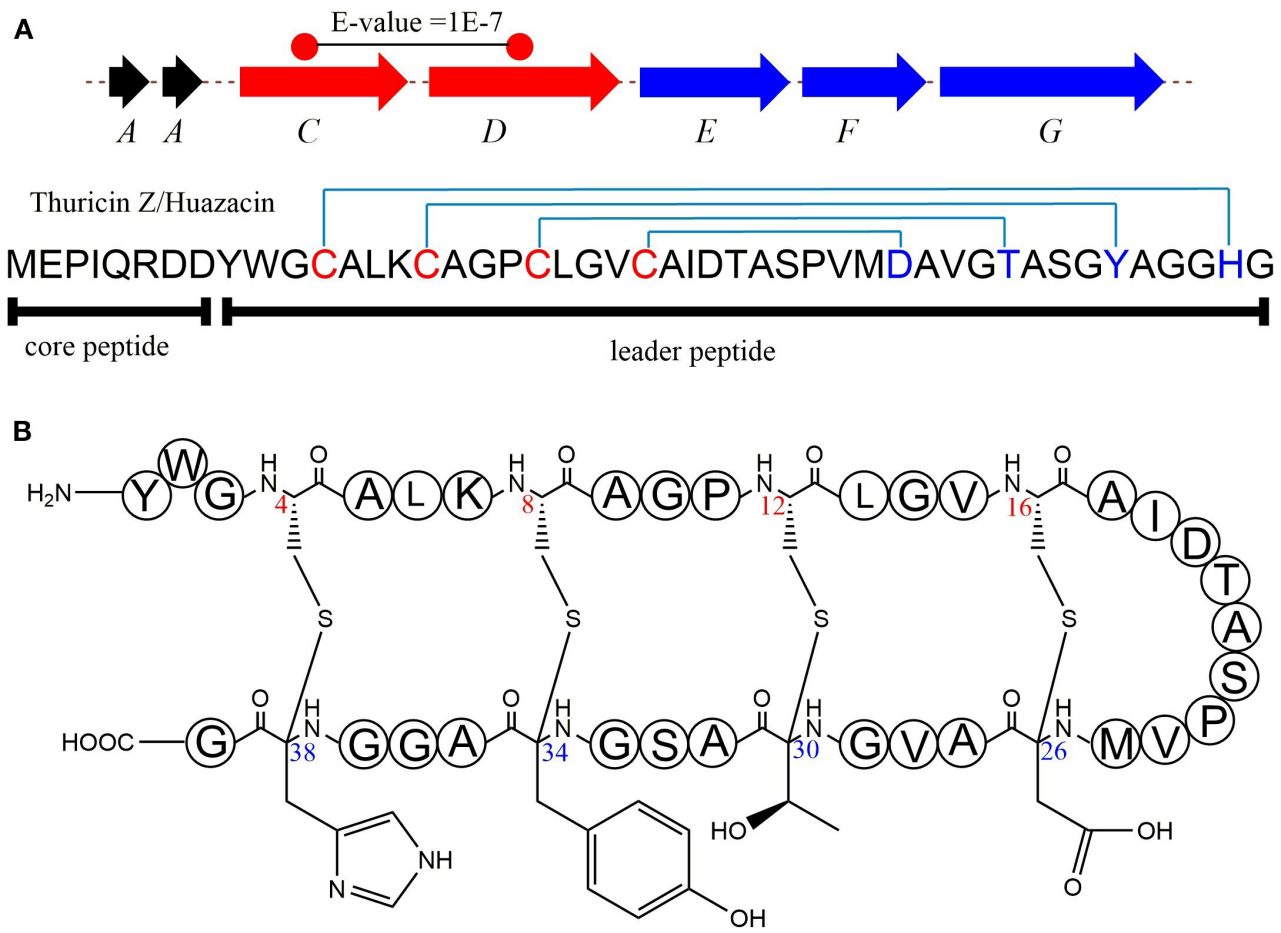
Biosynthesis and structural characterization of thuricin Z/Huazacin. **(A)** Organization of the *Thz* gene cluster. **(B)** Structure of thuricin Z or Huazacin (Mo et al., 2019).

In 2019, Mo et al. identified a new sactipeptide Thuricin Z in *Bacillus thuringiensis* serovar *huazhongensis* (Mo et al., 2019). The *thz* gene cluster is composed of seven genes. They are genes encoding 2 copies of the precursor peptide ThzA, two genes encoding putative rSAM enzymes ThzC and ThzD, *thzF* encoding a permeability enzyme, *thzE* and *thzG* encoding two ABC transporters (Figure 5A). It is noticeable that the *thz* gene cluster contains two precursor peptide genes and two rSAM synthetase genes, which is very similar to the thuricin CD gene cluster *trn* (Figure 2). There are two copies of genes *thzA* encoding two identical precursor peptides in the *thz* gene cluster, but distinct precursor peptide genes in thuricin CD gene cluster. The alignment BlastP e-value between the two rSAM enzymes ThzC and ThzD is 1E-7. This finding also raises an interesting question as to what are the functions of these two distant enzymes in modifications of the same precursor peptide. Subsequently, *in vitro*, experiments showed that both ThzC and ThzD could independently catalyze the formation of sactionine in the same way (Mo et al., 2019). The positions of the four thioether bonds of mature Thuricin Z are Cys16 to Asp26, Cys12 toThr30, Cys8 to Tyr34, Cys4 to His38, and all of the acceptor amino acids are D-configurations (Figure 5B). The formation of sevral S-Cα thioether bonds proceeds in the direction from the N-terminus to the C-terminus, which is similar to the sequential order in the RumC biosythesis mentioned above (Balty et al., 2019). In the same year, Mitchell et al. reported the same sactipeptide isolated from the medium extract and named huazacin (Hudson et al., 2019), whose chemical structure is coincident with Thuricin Z.

### GggB in Streptosactin Biosynthesis

**Figure 6:**
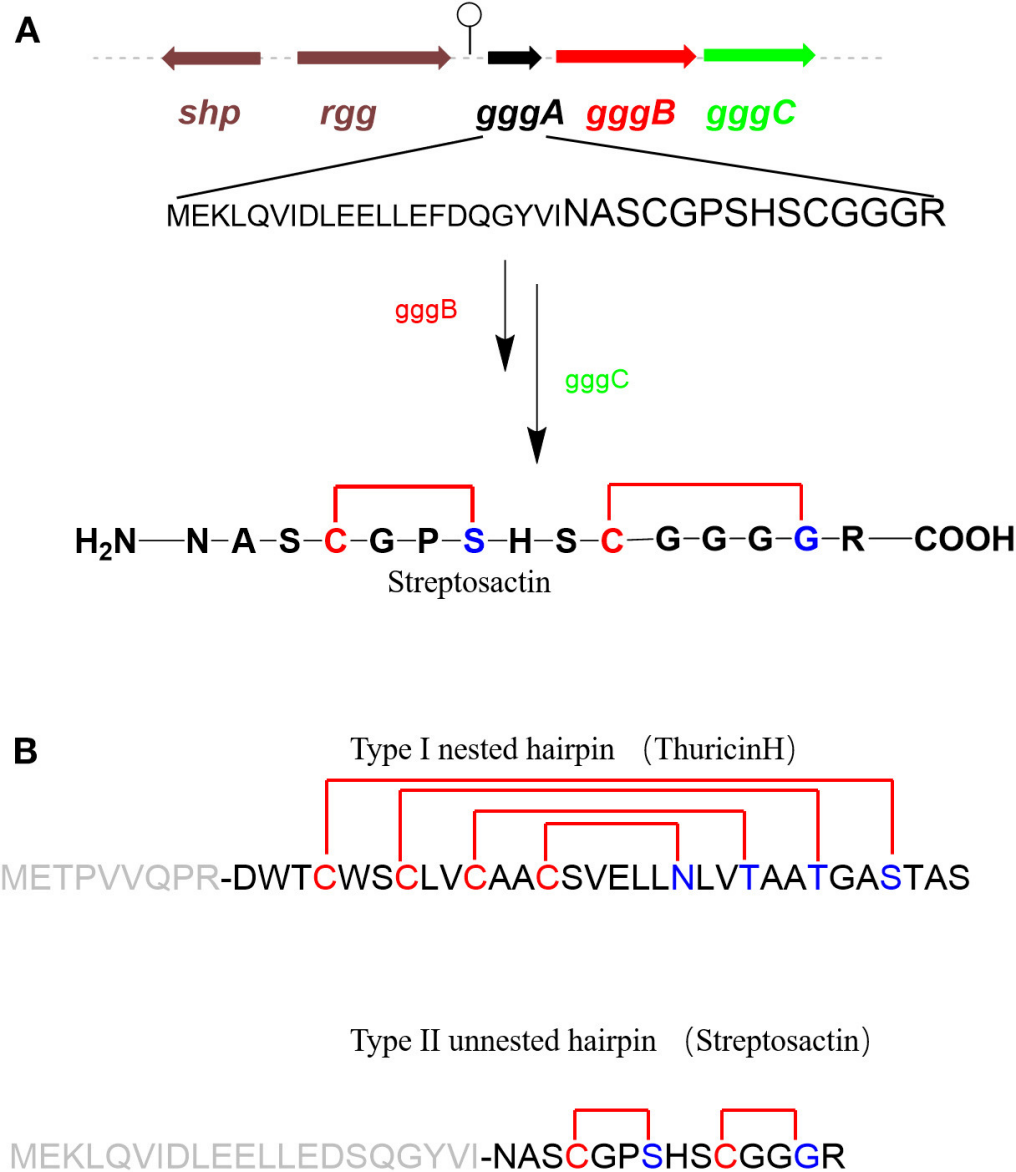
rSAM enzyme-modified RiPPs in *streptococci* (Bushin et al., 2020). **(A)** Biosynthesis gene cluster of streptosactin encoded by a precursor peptide (black), a rSAM enzyme (red), a protease (green), and regulators (brown). Hypothetical biosynthetic pathway of streptosactin. GggB installs two thioether bonds in the core region of GggA (black, majuscule), then the protease GggC, removes the leader (black, lowercase), releasing the mature 14 mer product streptosactin. **(B)** Comparison of the topology of known type I nested hairpin sactipeptides, with thurincin H as a representative example, and the new type II unnested hairpin streptosactin. The donor amino acids are yellow; acceptor amino acids are blue, leader sequences are gray.

Mammalian microbiomes encode thousands of biosynthetic gene clusters (BGCs). Among them, some encode ribosomally synthesized and post-translationally modified peptides (RiPPs) which are regulated by quorum sensing (Bushin et al., 2020) (Figure 6A). Bushin et al. mined out streptosactin in mammalian microbiome streptococci, which is the newestly characterized member in the growing list of sactipeptides to date. Streptosactin and ruminococcin C are the only two sactipeptides discovered in the human microbiome. In consideration of deficient production in the native host, it is impossible to isolate and characterize the original structure of streptosactin. The structure of the mature streptosactin was determined by learning from the structure of modified precursor peptide GggA by co-expression with enzyme GggB. HRMS/MS analysis of collision-induced dissociation of GggB product showed that all y-ions spanning His29 and Gly34 lacked two protons, but those from N-terminus to Ser28 lacked four, implicating that Ser28 and Gly34 were modified. Conserved cysteines located upstream of Ser28 and Gly34 made us think of the thioether bond's formation. Furthermore, HR-MS/MS fragmentation pattern of the product also indicated the presence of sactionine linkages. Various isotope-labeled GggA catalyzed by GggB gave new NMR signals and different chemical shift compairing with unlabeled GggA as substrate. The 1D ^13^C and 2D DEPT-edited HSQC spectrums provide the evidence for thioether bonds formation between Cα of Ser28 and Gly34. Moreover, donor/acceptor amino acid substitutions in GggA suggest that a duo of α-thioether bonds are located between Cys31-Gly34 and Cys25-Ser28. The two sactionine linkages occur in a defined order from C- to N-terminus. The formation of the second is dependent on the presence of the first one, which has a similar pattern with the sequential order of sactionine formation in Ruminococcin C. The absolute configuration at Cα of Ser28, which is an acceptor amino acid in thioether bonds, remains to be defined. Streptosactin showed two unnested sactionine macrocycles, different from all other sactipeptides feature a nested hairpin topology (Figure 6B). Analysis of the gene cluster, GggB was investigated as a radical SAM enzyme to modify the precursor peptide for installing the thioether linkages in streptosactin biosynthesis. Bioinformatic dissection and EPR spectrum detection indicated GggB is a sactisynthase with a C-terminal SPASM domain. GggB was heterologously expressed and reconstituted under anaerobic conditions, subsequently *in vitro* reaction with precursor peptide GggA prepared by solid-phase peptide synthesis. GggB exhibites the same appearance that initial generation of the substrate Cα radical is a conservative process catalyzed by sactisynthase, but its real mechanism remains to be disclosed to date (Bushin et al., 2020).

## Activity and Antibacterial Mechanism of Sactipeptides

Except for the spore killing factor (SKF), the physiological functions of most of the sactipeptides in the producer strains remain unknown. SKF produced by the *skf* operon of *B. subtilis* 168 plays a key role in the spore-forming process. When the *B. subtilis* population experienced nutrition limitation, the regulatory protein Spo0A activated *skf* and *sdp* operons' expressions. Then the strain produced the SKF and the sporulation delay protein SDP, respectively. The extracellularly secreted compounds SKF and SDP began to lyse and released additional nutrients to *B. Subtilis* subpopulations, delaying their sporulation process (Engelberg-Kulka and Hazan, 2003; Gonzalez-Pastor et al., 2003; Liu et al., 2010). Thuricin CD, produced by *B. thuringiensis* DPC 6431, shows a narrow-spectrum antibacterial activity in killing the pathogen *Clostridium difficile* (Rea et al., 2010, 2011; Mathur et al., 2016). Further research demonstrates that thuricin CD can disrupte the cell membrane of bacteria, and this situation cannot be reversed even under the condition of membrane potential repolarization. In addition, the thuricin CD's depolarizing effect on the cell membrane changes the size, particle diameter, and physiological morphology of the bacterial cells. Therefore, speculated antibacterial mechanism of thuricin CD is inserting into the target bacterial cell membrane to form channels, resulting in the permeability increasing and intracellular ions flowing out; this action causes the cell membrane to depolarize and factually cell dead (Mathur et al., 2017). The sactipeptide thuricin Z produced by *B. thuringiensis* serovar *huazhongensis* also exerts its antibacterial activity by acting on the cell membrane, causing the permeability changing of the target cell membrane (Mo et al., 2019). Subtilosin A, produced by *B. subtilis* 168, shows broad-spectrum activity against both gram-positive and gram-negative bacteria, including aerobic and anaerobic bacteria (Shelburne et al., 2007). The T6I mutant of Subtilosin A also shows hemolytic activity (Huang et al., 2009). It is hypothesized that the antibacterial activity of subtilosin A is depended on its hydrophilic and hydrophobic surfaces which can interfere and disrupt the bilayers of phospholipids (such as cell membranes) (Thennarasu et al., 2005). In addition, subtilosin A also exhibited spermicidal activity (Silkin et al., 2008; Sutyak et al., 2008). Thurincin H produced by *B. thuringiensis* SF361 is effective against *Bacillus* spp., *Micrococcus* spp., *Listeria* spp, such as *L. monocytogenes* which are the main pathogens of human listeriosis (Lee et al., 2009; Sit et al., 2011b; Wang et al., 2014c). Simultaneously, thurincin H can be used as a food preservative for the control of food originated Gram-positive bacteria that cause the deterioration of sunflower honey (Lee et al., 2008, 2009). Unlike some other sactipeptides, such as Subtilosin A and thuricin CD which act on cells membrane, in 2014, Wang et al. confirmed that thurincin H caused morphological changes in target bacteria, not by affecting changes in cell membrane permeability but by targetting other receptors (Wang et al., 2014a). In 2019, Benjdia et al. reported that the sactipeptide RumC1 andC2 produced by human gut symbiont *Ruminococcus gnavus* are against Gram-positive bacteria *Clostridium perfringens, Bacillus subtilis*. Intriguingly, the RumC1 can also induce a lag phase for Gram-negative bacteria *Escherichia coli* (Balty et al., 2019). The antimicrobial activities of RumC against both Gram-positive and Gram-negative bacteria indicate that RumC may target an intracellular target but not the cell envelope (Balty et al., 2019). In the same year, Duarte et al. also reported the molecule RumC1 again (Chiumento et al., 2019). Their results showed that RumC1 also has a broad-spectrum antibacterial activity. RumC1 remarkably resists various pathogenic bacteria and multi-drug resistant bacteria by affecting bacteria's nucleic acid synthesis. The measured MICs are equivalent to vancomycin. Further preclinical studies have shown that RumC1 could cure infections in the mammalian. It also encompass some properties such as safe for gut tissue, stability by oral delivery. Therefore, RumC1 has essential features of a drug candidate and has promising prospect to be a new drug against resistant anaerobic and aerobic clinical pathogens (Chiumento et al., 2019; Roblin et al., 2020). The newest sactipeptide streptosactin reveals potent but narrow-spectrum activity against its producing strain. Interestingly, it also inhibits its closest relatives carrying the same RiPP BGCs. A series of self-killing activity detections and even the cell clumping phenotype suggested streptosactin may be the first fratricidal agent from *Streptococcus thermophiles* (Bushin et al., 2020).

## Distributions of Sactipeptides

Thus far RiPPs are found in all three domains of life and exhibit diverse structures and bioactivities, representing a promising area of chemical and genetic space for natural product exploration. The biosynthetic gene cluster of RiPPs generally comprises a precursor peptide gene and a modest number of promiscuous modification enzyme genes (Montalban-Lopez and Kuipers, 2016). Because of its ribosomal origin, RiPPs offer an effective approach to interrogate the structure-activity relationship in RiPP maturation and to generate structural variants by precursor peptide bioengineering. The heterologous expression systems were successfully established in *E. coli* and *Streptomyces* to produce RiPPs. Mutagenesis studies of precursor peptide were carried out to not only obtain valuable insights into RiPPs maturation and the substrate tolerance of relevant enzymes, but also create a large variety of bioactive molecules employing with enhanced antimicrobial activity (Mo et al., 2017; Ma and Zhang, 2020). Benefited from rapid progress on DNA sequencing technology, *in silico* mining of genomes combined with molecular biology approaches has guided the discovery of a large number of new RiPPs natural products. In general, various algorithms, including antiSMASH, RiPPMiner, BAGEL4, RiPPER, and RiPP-PRISM can be used for mining the RiPP BGCs (Velasquez and Van der Donk, 2011; Poorinmohammad et al., 2019; Russell and Truman, 2020; Zhong et al., 2020). The most important characteristic of sactipeptide biosynthesis is the presence of radical SAM enzyme sactisynthase, which will direct the promising way for genome-mining discovery of sactipeptide. Since 1985, subtilosin A as the first sactipeptide was discovered from *Bacillus subtilis* 168 (Babasaki et al., 1985). More than 10 years later, four other sactipeptides were found and identified. These sactipeptides derived from the aerobic *Bacillus* spp. are sporulation killing factor (SKF) (Liu et al., 2010), thurincin H (Lee et al., 2009; Sit et al., 2011b) and thuricin CD (a class of two-component sactipeptides) (Rea et al., 2010; Sit et al., 2011a) (Figure 2). With the advancement of bioinformatics methods and genomic sequencing technology, more and more sactipeptides have been discovered and identified in recent years. In 2014, Hertweck et al. utilized anti-SMASH (Lee et al., 2008; Wang et al., 2014c; Lee and Lee, 2016), BAGEL3 (Wang et al., 2014b), BLAST and other bioinformatics tools to perform RiPPs genome mining on anaerobic bacteria with known genome sequences. The results demonstrated that 59 of 211 strains (28%) contain the RiPPs gene cluster. Further analysis of the 81 RiPPs gene clusters in the 59 strains revealed 16 sactipeptide gene clusters from Proteobacteria and one from Thermotogae. The remaining 14 sactipeptides were distributed in Firmicutes, and 8 of the 14 sactipeptides were distributed in *Clostridium* (Azevedo et al., 2015). These studies indicate that the previously discovered sactipeptides are mainly distributed in two main species of Firmicutes: aerobic *Bacillus* and anaerobic *Clostridium*. In 2015, Azevedo et al. carried out genome mining studies on the distribution and diversity of bacteriocin on 224 ruminal bacterium and five ruminal archaea.

**Figure 7:**
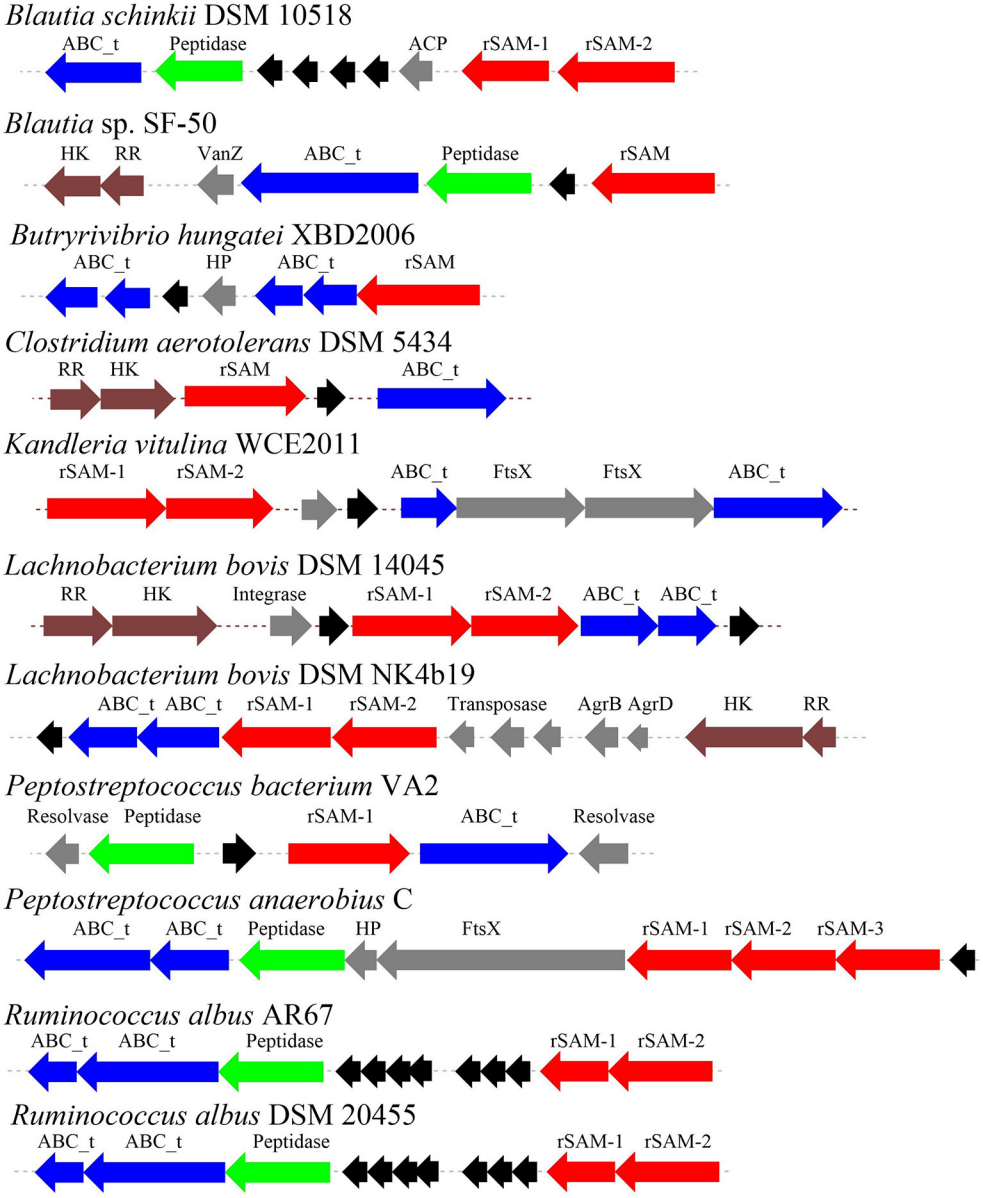
The biosynthetic gene clusters of the sactipeptides of ruminal bacteria were discovered by Azevedo et al. (2015). Blue, genes involved in transport; black, structural genes; red, rSAM; green, protease proteins; gray, undefined proteins.

**Figure 8:**
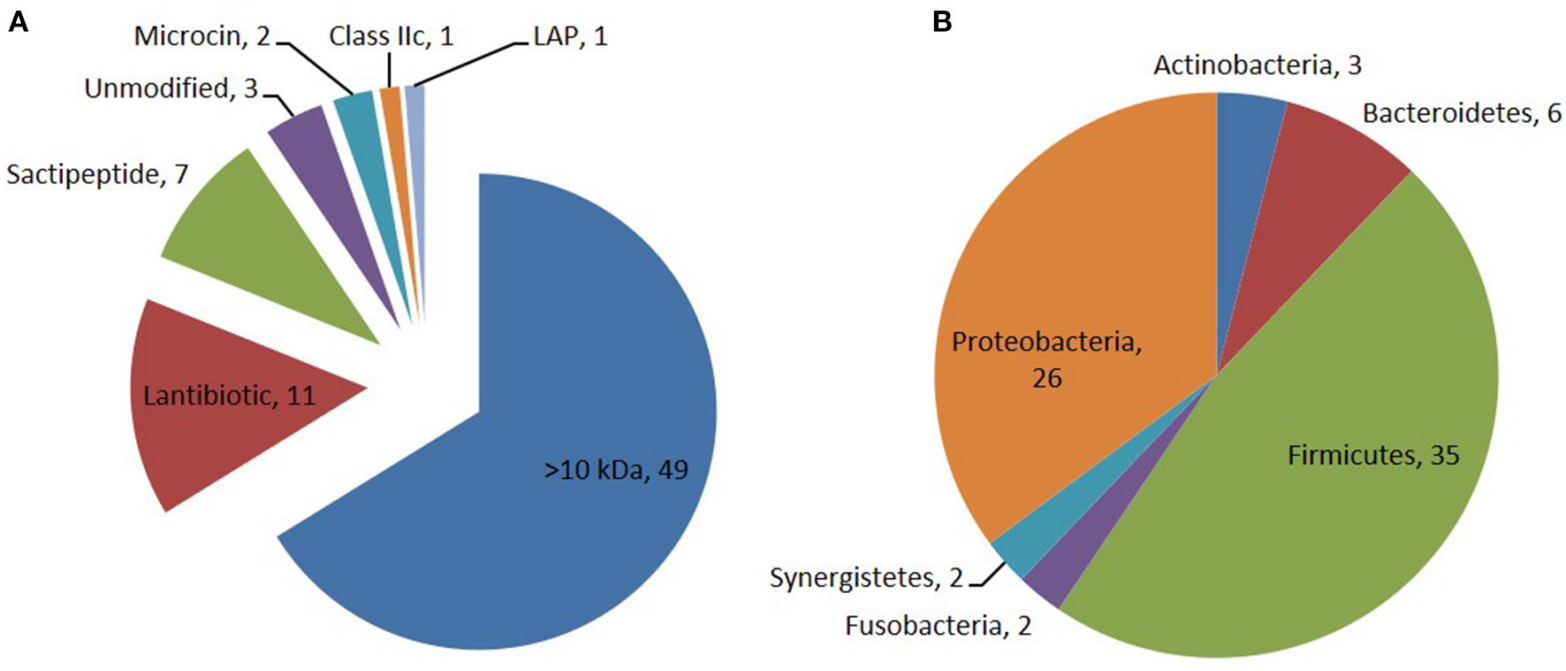
Distribution of bacteriocin class **(A)** and their producing phylum among the 74 PBGCs **(B)** identified by Walsh et al. (2015). Seven sactipeptide gene clusters [(green triangle in **(A)**] were identified in 74 bacteriocin gene clusters which are mainly distributed in proteobacteria [brown triangle in **(B)**] and Frimicutes [green triangle in **(B)**].

**Figure 9:**
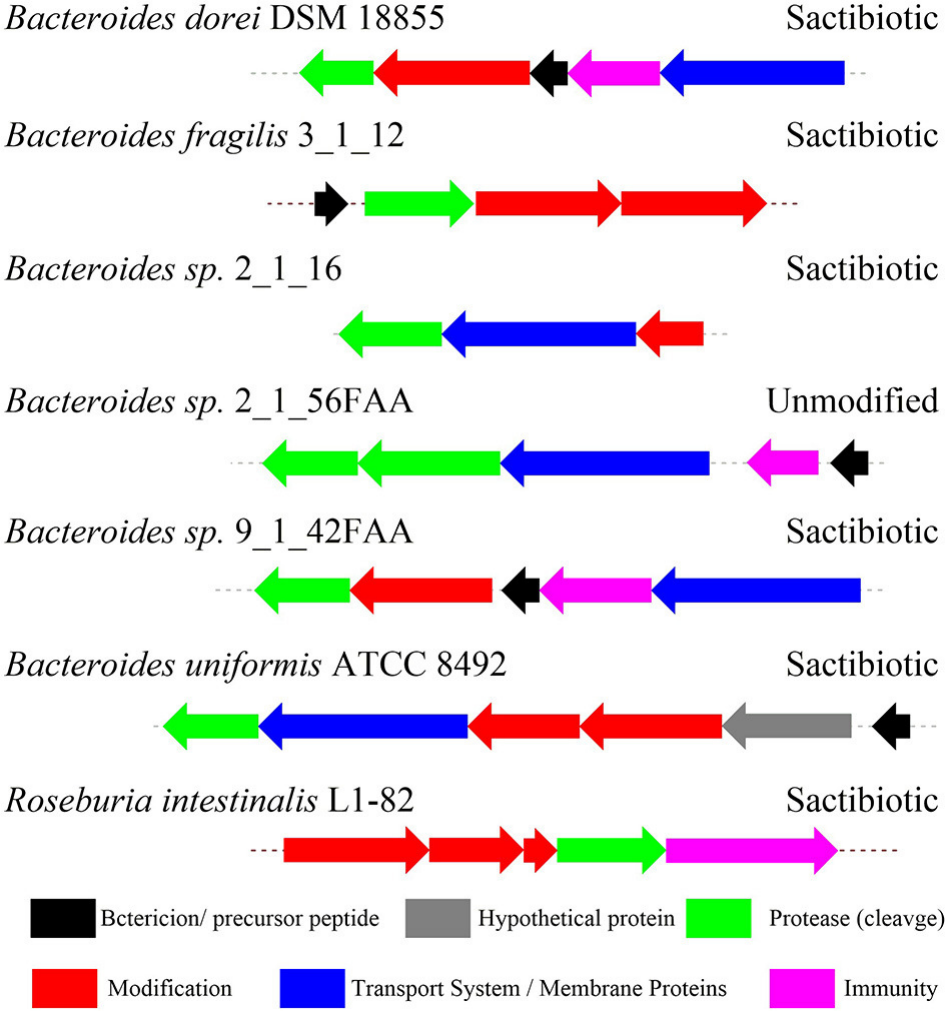
The biosynthetic gene clusters of the sactipeptides in genome-sequenced isolates from the gastrointestinal tract identified by Walsh et al. (2015).

Through the primary bioinformatics analysis, 51 genomes among 229 ruminal microorganisms contain 68 putative sactipeptide gene clusters. The clusters usually take ABC transporter and rSAM protein in the 20-kb region adjacent to the precursor peptide on the genome as selective markers. Further analysis suggested that there are 11 sactipeptide clusters from different bacterial genomes with a size from 5.1 kb (*Butyrivibrio hungatei*XBD2006) to 10.1 kb (*Peptostreptococcus anaerobius*C) (Figure 7) (Azevedo et al., 2015). In the same year, Walsh et al. analyzed the genomic data of the American microbiome project. They identified 59 unique Fusobacteria and Synergistetes, 74 bacteriocin gene clusters from the human gut microbiota, which included seven sactipeptide gene clusters (Figures 8, 9). The above research further revealed the broad distribution of sactipeptides among different microbial species (Walsh et al., 2015).

## Other Radical Sam Enzymes Catalyze Sulfur-to-non-α-Carbon Thioether Bonds Formation

### QhpD Involved in the Formation of Sulfur-to-Methylene Carbon Thioether Bonds

**Figure 10:**
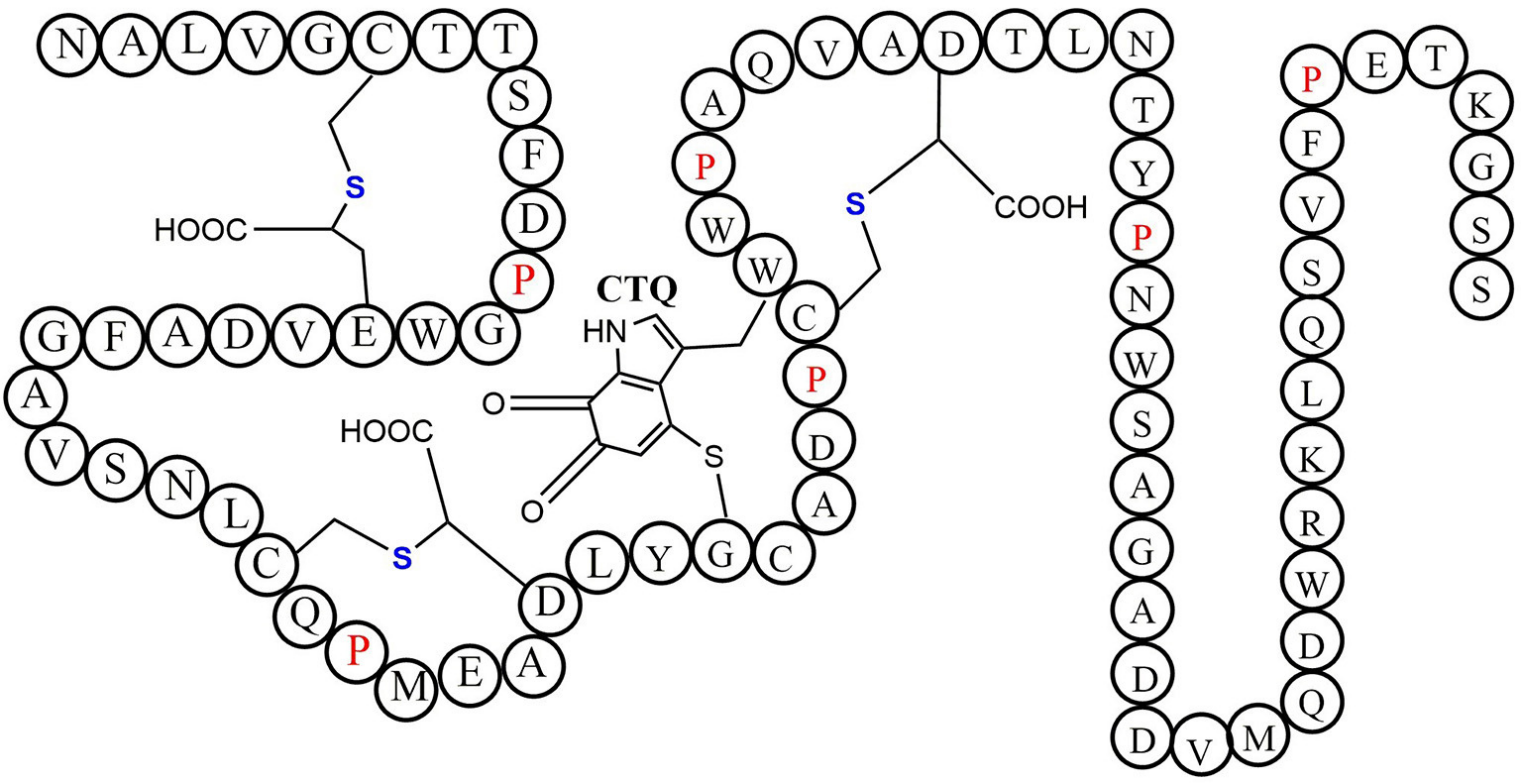
The schematic structure of cofactor-cysteine tryptophylquinone (CTQ) is shown with the thioether bonds' stereochemistry and the amino acid residues in a single-letter code (Nakai et al., 2014).

Quinohemoprotein amine dehydrogenase (QHNDH) is an enzyme that catalyzes oxidative deamination of various aliphatic primary amines to provide energy, carbon, and nitrogen sources (Takagi et al., 1999, 2001). Although the enzyme QHNDH was initially discovered in a limited number of Gram-negative bacteria (Adachi et al., 1998; Takagi et al., 2001), some recent investigations revealed a wide distribution of the *qhp* operon encoding enzyme QHNDH in both Gram-negative species and Gram-positive bacteria (Nakai et al., 2014). QHNDH usually consists of three different subunits and exhibits distinct structural features (Datta et al., 2001; Satoh et al., 2002). The *qhpA* gene encodes the biggest 60 KD subunit α which has four domains with two hemes which mediate electron transfer from the substrate donor to an external electron acceptor (Adachi et al., 1998; Takagi et al., 2001). The *qhpB* gene encodes the medium-sized subunit βof 36 kDa protein with a highly conserved β-propeller structure in quinohemeprotein dehydrogenases. The small ~9 kDa subunit γ encoded by *qhpC* has a unique structure with four internal thioether bonds (Figure 10). The sulfur atom of the Cys37 residue crosslink to the indole group of the Trp43 residue. In the meantime, The indole group of tryptophan is distinctively oxidized to an *ortho*-quinone unit and form a peptidyl quinone cofactor-cysteine tryptophylquinone (CTQ). The other three cysteines (Cys7, Cys27, Cys41) crosslink to the methylene carbon atom of glutamine or Aspartic acid residues (Glu16, Asp33, Asp49). These three cross structures support the structural stabilization of the polypeptide chain. Therefore, a set of enzymes encoded by genes *qhpD, qhpE, qhpF*, and *qhpG* involved in the *qhp* operon modifying the translational γ-subunit peptide (Ono et al., 2006; Nakai et al., 2012, 2014). Post-translational modifications for γ-subunit of QHNDH occur independently or at the condition of associating with the α and β subunit peptides. All three mature subunits finally constitute the active QHNDH enzyme complex. The bacterial rSAM enzyme QhpD is essential for the maturation of QHNDH dehydrogenase. It participates in the formation of intra-protein thioether bridges within the small subunit γ to give maturated QhpC. Tsubaki et al. heterogeneously overproduced an unsoluble protein enzyme QhpD originating from *Paracoccus denitrificans* (Nakai et al., 2015). Full-wavelength absorption scan, EPR spectra detection, and iron and sulfur contents measurement indicated that there are multiple (likely three) [4Fe-4S] clusters in the reconstituted QhpD. *In vitro*, in the presence of SAM, the QhpD reduced by sodium dithionite can catalyze reductive cleavage of SAM into methionine and 5-deoxyadenosine and subsequent the single or multiple thioether bonds formations between the sulfur atom and the carbon atom of methylene in QhpC. The possible reaction mechanism for QhpD is similar to AlbA involved in the construction of the sactionine. These findings will provide the fundamental theories for the rSAM enzymes acting on a ribosomally translated large protein distinguish from the usual precursor peptide as substrate (Nakai et al., 2015).

### Ranthipeptide (Radical Non-α-carbon Thioether Peptides), Thermocellin and Freyrasin

**Figure 11:**
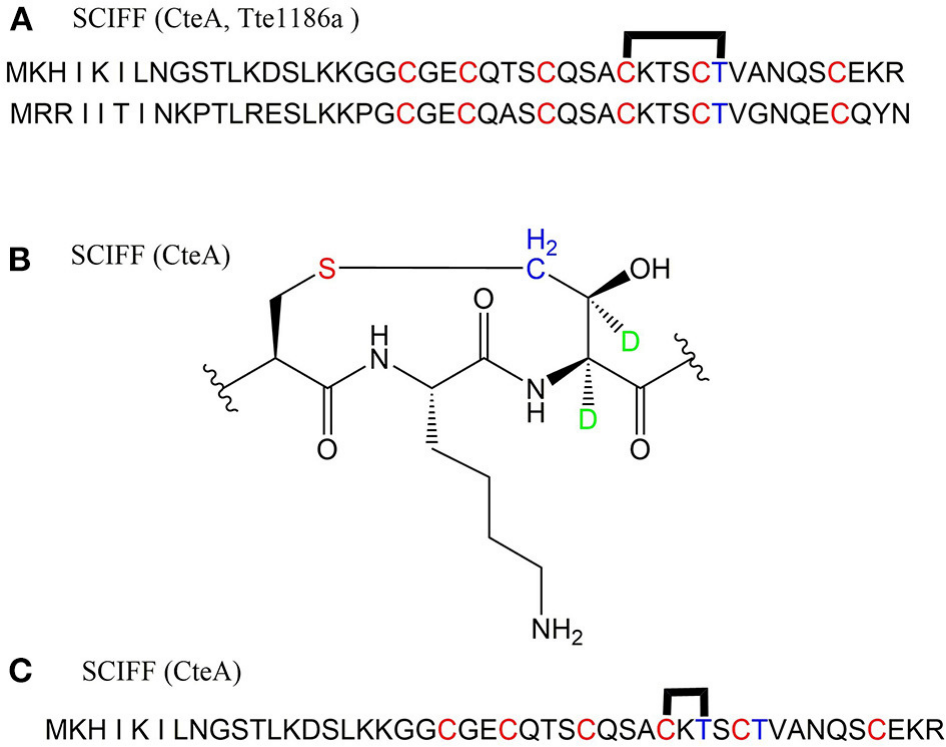
Structural characterization of SCIFFs. **(A)** Previously characterized thioether bonds intramolecular of CteA and Tte1186a. **(B)** Recent studies demonstrate the thioether bond was formed between thiol of Cys and γ-carbon of anther Thr in CteA. **(C)** Positions of amino acids involved in thioether bond formation in heterologous expression systems (Grove et al., 2017; Hudson et al., 2019).

**Figure 12:**
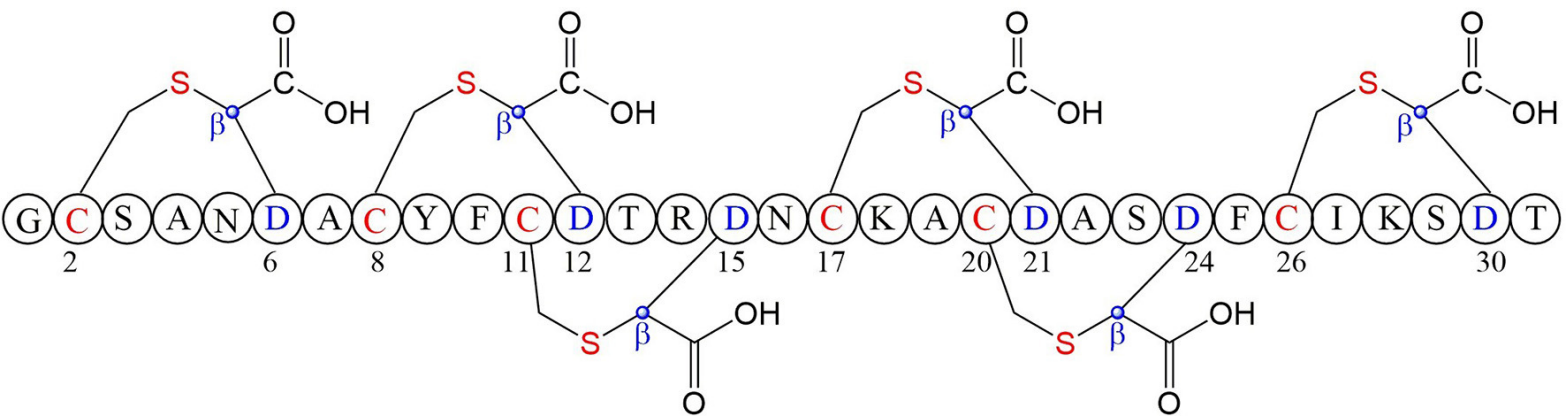
Structure of freyrasin the donor amino acid and thioether marked with red, the acceptor amino acid and its β-carbon atom marked with blue (Hudson et al., 2019).

A large class of unique precursor peptides named SCIFF (six cysteines in 45 residues) were found in *Clostridia*, during bioinformatic searching for rSAM enzyme and its targeted post-translationally modified precursor peptides. Characteristics of this new class peptide is an average of 6 Cys residues in the conserved C-terminal 45 amino acids region of the precursor peptide (Haft and Basu, 2011). Tte1186a is the first identified SCIFF peptide. In 2016, Bandarian et al. cloned SCIFF precursor peptide gene *tte1186a* and its modified enzyme gene *tte1186* from *Caldanaerobacter subterraneus* subsp. *tengcongensis* MB4. Subsequent *in vitro* catalytic assays combined with mass spectrometry identification experiments showed that rSAM enzyme Tte1186 could catalyze the formation of thioether bond between Cys32and Thr37 of the precursor peptide encoded by *tte1186a* (Figure 11A) (Bruender et al., 2016). Thermocellin was the first SCIFF-like peptide reported in *Clostridium* (Grove et al., 2017). In 2017, Bowers et al. reported the thermocellin from *Clostridium thermocellum*. The C-terminal region of its precursor peptides CteA has high sequence similarities with Tte1186a. *In vitro* catalytic experiment of CteA by rSAM enzyme CteB combined with mass spectrometry analysis showed that the number and position of the thioether bonds formed inside the CteA molecule were utterly consistent with Tte1186a (Figure 11A) (Grove et al., 2017). However, recently, Mitchell et al. conducted a co-expression experiment of CteB and CteA in *E. coli*. The hydrogen atoms of Cα and Cβ positions of Thr residues in CteA were deuterated and Cγ position was unlabeled (1H). Mass spectrometry showed that the molecular weight of the modified CteA was reduced by 2Da instead of 3Da compared to the original substrate, clearly indicating that the thioether bond formation between Cys and Cγ position of Thr in CteA molecule (Figure 11B). Besides, Mitchell et al. also found that the amino acids for the formation of thioether bond were Cys32 and Thr34 in a heterologous expression system (Figure 11C), inconsistent with the previously reported results. Since the previous results concluded from *in vitro* catalytic experiments, it could not rule out whether this difference was attributed to different reaction systems between *in vivo* and *in vitro* (Hudson et al., 2019). Meanwhile, Mitchell et al. also reported another type of SCIFF named freyrasin from *Paenibacillus polymyxa*. Co-expression of the precursor peptide gene *papA* and rSAM enzyme gene *papB* (homologous to *qhpD*) in *E. coli*, combined with bioinformatic analysis, mass and NMR spectrometries, finally determined the freyrasin molecule contains six Cys-Asp (S-Cβ) thioether bonds (Figure 12). So far, a series of results indicate that SCIFFs are not in the category of sactipeptide; instead, SCIFFs thioether bonds are between the sulfur atom of donor cysteine and the β or γ-carbon atoms of acceptor amino acids. To better reflect its biosynthesis process dependent on rSAM enzymes and its difference to lanthipeptide (S-Cβ thioether bond), Mitchell et al. named this type of RiPPs as Ranthipeptides (Hudson et al., 2019). In the subsequent research, Mitchell et al. confirmed that the rSAM enzyme PapB could catalyze the complete generation of 6 (S–Cβ) thioether bonds on PapA *in vitro*. Moreover, the tolerance of rSAM enzyme PapB to the substrate was tested using single mutations of the six donors cysteines and six acceptor aspartic acids. The results showed: When each Asp in PapA was mutated to Ala individually, compared with the wild type, the mutation position could not form a thioether bond with the corresponding Cys, the other five Cys–Asp (S–Cβ) thioether bonds formed normally in the precursor peptide; When each Asp in PapA was individually mutated to Asn, the result is same as above, which is comprehensively indicating that the side-chain carboxyl group of the acceptor amino acid could be necessary for the recognition by the modified enzymes; When each Asp in PapA was mutated to Glu individually which has a similar topology structure as the Asp, and the six thioether bonds can be completely formed, which are five Cys–Asp (S–Cβ) and one Cys-Glu (S–Cγ) thioether bonds. These results further confirm that the side chain carboxyl of the acceptor amino acid is essential for the recognition by modified enzymes to form the thioether bonds. Under natural conditions, Cys-X3-Asp of PapA is the recognition motif for rSAM enzyme PapB; when an amino acid residue was added to this motif (Cys-X4-Asp), the rSAM enzyme PapB could not catalyze the formation of thioether bond anymore. When one amino acid residue was deleted (Cys-X2-Asp), mostly, the rSAM enzyme PapB was not able to catalyze the formation of the thioether bond on this motif. These indicate that the rSAM enzyme PapB recognizes catalytic substrate with Cys-X3-Asp strictly (Grove et al., 2017).

## Application Prospects of Sactipeptide Natural Products

In the past 20 years, biologists and chemists have carried out systematic and in-depth research on sactipeptides, including structural elucidation, activity testing, and biosynthesis mechanism uncovering. As peptide macromolecules, sactipeptide possess multiple application prospects in many aspects, such as biological control, food preservation, clinical treatments. For example, some sactipeptides have significant inhibitory activity against human multi-drug resistant pathogenic bacteria. Due to their narrow antibacterial spectrum, they are harmless to mammalian cells, not leading to drug resistance and hence showing good potential to develop a new clinical antibacterial agent. It is necessary to develop environmentally friendly, safe and better biocompatible sactipeptide preservatives (such as thuricin H separated from honey and RumC produced by human gut symbiotic microorganisms) to replace chemical preservatives (such as sodium benzoate or potassium sorbate, etc.). In recent years, the agricultural departments have vigorously advocated reducing chemical fertilizers and pesticides with bio-organic fertilizers and active biological microorganisms, such as sactipepetide producer *Bacillus thuringiensis* first live *Bacillus* bacteria used in agricultural biological control. This improvement will optimize the ecological environment and reduce pesticide residues of farm products. On the other hand, many old antibiotics such as macrolide antibiotics and organophosphorus pesticides are widely used in agriculture. They are difficult to be degraded by the environment. The accumulation of pesticides has severe ecological hazards. However, sactipepetide is a ribosomally synthesized and post-translationally modified peptide. As a polypeptide compound, its skeleton consists of proteinogenic amino acids, facilitating the degradation in the body. These advantages make sactipeptides be enormous potential for developing as novel green pesticides in the future. All in all, the progress of studies on sactipepetide will favor various fields such as agriculture, food, medical, and health.

## Author Contributions

YC, JW, and WD wrote the manuscript. GL and YY drew most of the figures. All authors contributed to the article and approved the submitted version.

## Conflict of Interest

The authors declare that the research was conducted in the absence of any commercial or financial relationships that could be construed as a potential conflict of interest.
